# Supplementary material for: Treatment failure to sodium stibogluconate in cutaneous leishmaniasis: A challenge to infection control and disease elimination
Source: PLoS One. 2021 Oct 22;16(10):e0259009. doi: 10.1371/journal.pone.0259009 (PMC8535432; doi:10.1371/journal.pone.0259009)
Supplement: S1 Table — (PDF) [file pone.0259009.s001.pdf]

S1 Table: Binary logistic regression conducted with varying conditions and the respective results

	Analysis	Result
1	Binary logistic regression with 'Enter' model selection criteria, for all the variables in their natural form	At 5% level of significance, none of the variables were statistically associated with response to treatment
2	Binary logistic regression with 'Backward Stepwise - Wald' as model selection criteria, for all variables in their natural form	Coefficients of variables in all steps, except for the coefficient of *Site 2* in Step 5 (p-value = 0.044) and *Type 1* in Step 10 (p-value = 0.028) were not significant.<br><br>Order of the variables removed from the model in each step was: *Type 3* → *Type 4* → *Age* → *Site 1* → Gender → *Site 4* → Time Since Onset → *Site 3* → *Type 2* → *Site 2*.<br><br>The variables, *Number of lesions* and *Type 1* were not removed and remained in the final model. The p-values were 0.053 for *Type 1* and 0.064 for *Number of lesions.*
3	Binary logistic regression with 'Backward Stepwise - Wald' as model selection criteria was repeated 3a) using *Time Since Onset* as categories, with other variables in their natural form and 3b) *Age* as categories, with other variables in their natural form.	The explanatory power of predictor variables improved with both categorisations, resulting in a higher coefficient of determination (R^2 value).<br><br>Thus, the 'Final regression model' was formulated as a binary logistic regression with backward Wald, using the variables, categorised *time since onset (TSO)*, categorised *age*, other variables in their natural form and taking treatment failure as the base category.
4	'Final regression model' was fitted for the whole data set	A model equation could not be formulated for the response variable 'Response to SSG' as there were no statistically significant coefficients.<br><br>However, the variables *Number of Lesions* (p-value=0.064) and *Type 1* (p-value=0.053) persisted in the final model, with the signs of the coefficients indicating that there is a positive relationship between them and the response to SSG.<br><br>Furthermore, the variable *Site 2* was statistically significant in four steps in the model building process and it can be said that there is an impact of the lesion site *Site 2* (trunk) to the Response to SSG. When considering the sign of coefficient of *Site 2* in all models built in each steps, it can be seen that the sign is negative. Therefore, the relationship of Lesion site *Site 2* with response to treatment is negative, i.e., a lesion on *Site 2* (trunk) was more prone for TF.
5	'Final regression model' was fitted to three age categories separately, where the classes were approximately balanced: 1-30years (65 cases), 31-50years (75 cases) and 51 to70 years (61 cases).	**Age category 1 to 30:**<br>The coefficients associated with the variables *Number of Lesions*, *Site 1*, *TSO 1*, *TSO 2* and *TSO 3* were significant at 5% level of significance in the final model (p-value < 0.05).<br><br>The equation of the fitted model:<br><br>$\log\left(\frac{p}{1-p}\right) = -1.714 + 2.105(NoOfLesions) + 1.825(Site1) - 3.063(TSO1) - 2.535(TSO2) - 3.551(TSO3)$<br><br>A positive relationship was found among the variables *Number of Lesions* and *Site 1* with Response SSG. The three variables *TSO 1*, *TSO 2* and *TSO 3* are negatively related to the variable *Response to SSG*.<br><br>**Age category 31 to 50 years:**<br>No variables were preserved in the final model. It is not possible to write a model equation as all the coefficients were not significant in last few steps.<br><br>*Type 2* is the variable removed from the model in the last step. In the Step 15, the coefficient of the variable *Type 2* is a negative value.<br><br>The coefficients of the variables *Site 2* and *Site 3* are significant in three steps while those of *Site 4* is significant in two steps, with all negative values, resulting in a negative relationship with *Response to SSG.*<br><br>*TSO 1* is significant in one step and the variable removed at one before the last step is *TSO 1* with a positive coefficient value.<br><br>**Age category 51 to 70 years:**<br>No variables were preserved in the final model and all the coefficients were not significant in last few steps. *TSO 1* is removed from the model in the last step and *TSO 2* in the step before the last, with positive coefficients. Thus, the variables *TSO 1* and *TSO 2* have an impact on Response to SSG in this age category.
