# Supplementary material for: Treatment failure to sodium stibogluconate in cutaneous leishmaniasis: A challenge to infection control and disease elimination
Source: PLoS One. 2021 Oct 22;16(10):e0259009. doi: 10.1371/journal.pone.0259009 (PMC8535432; doi:10.1371/journal.pone.0259009)
Supplement: Attachment — Submitted filename: Response to Reviewers.pdf [file pone.0259009.s003.pdf]

<u>Itemized list of specific responses</u>

PONE-D-21-23044
Treatment failure to sodium stibogluconate in cutaneous leishmaniasis : A challenge to infection control and disease elimination
PLOS ONE

Please find below the itemized list of specific responses to each of the comments (**page and line numbers refer to the unmarked version of the revised manuscript without tracked changes - the file labeled 'Manuscript'**).

<u>[1] Journal requirements:</u>
1) Please ensure that your manuscript meets PLOS ONE's style requirements, including those for file naming.

Response: PLOS ONE style template was referred, and the manuscript has been revised accordingly.

2) We note that Figure 1 in your submission contain map/satellite images which may be copyrighted

We require you to either (1) present written permission from the copyright holder to publish these figures specifically under the CC BY 4.0 license, or (2) remove the figures from your submission:

Response: Removed the Figure 1 from the manuscript since GPS coordinates have been included in the manuscript (page no: 5, line no: 116-117)

3) Please include captions for your Supporting Information files at the end of your manuscript, and update any in-text citations to match accordingly.

Response: Supporting information file renamed as 'S1 Table' , caption is at the end of the manuscript and in-text citations matched accordingly.

<u>[2] Editor Comments:</u>
Antimony-based anti-leishmanial treatment has been in use for many decades. Such prolonged use leads to resistance of the target pathogen to the anti-leishmanial. This situation can become grave with lack of appropriate control policies.

While the manuscript deals with an important issue in Leishmania treatment, it requires a few modifications as raised by the reviewers. Please include the different mechanisms of the antimony resistance published till date in the form of a table. Another Reviewer also suggested to provide a table on prevalence, parasites and disease profiles including serological profiles. A comparison between CL and VL for antimony-resistance will be a good guideline for the readers.

Response:

1) Authors agree

2) Causative parasites, prevalence, clinical profile and serological diagnosis of the main disease forms are given as a table (Table 4). Comparison between CL & VL for antimony resistance is given in the manuscript text (page no: 16, line no: 243-252)

3) Different antimony resistance mechanisms are now summarized in table 5.

[3] Review comments to the Author:

Reviewer #1: This is an interesting paper on Treatment failure to sodium stibogluconate in cutaneous leishmaniasis.
The study is well designed with sound analysis. However, the manuscript would be strengthened by addition of a table on clinical serological parameters.

Response: Authors agree . Table 4 has been added with the relevant information.

Reviewer #2: The efforts taken by authors to report an crucial health issue in Shrilanka is appreciated. However study needs to be more thorough with respect to the molecular mechanisms associated with drug resistance which will help to discover the drugs that could be used to treat SSG resistant Leishmania strains.

Response: Authors agree. The relevant information has been added in table 5 and described in the manuscript on page: 16, line: 252-256
